# Targeted Molecular Therapies in the Treatment of Esophageal Adenocarcinoma, Are We There Yet?

**DOI:** 10.3390/cancers12113077

**Published:** 2020-10-22

**Authors:** Shayan Khalafi, Albert Craig Lockhart, Alan S. Livingstone, Wael El-Rifai

**Affiliations:** 1 Department of Surgery, Miler School of Medicine, University of Miami, Miami, FL 33136, USA; sxk1000@miami.edu (S.K.); alivings@med.miami.edu (A.S.L.); 2 Department of Medicine, Miler School of Medicine, University of Miami, Miami, FL 33136, USA; aclockhart@med.miami.edu; 3 Sylvester Comprehensive Cancer Center, Miler School of Medicine, University of Miami, Miami, FL 33136, USA; 4 Department of Veterans Affairs, Miami Healthcare System, Miami, FL 33136, USA

**Keywords:** esophageal adenocarcinoma, targeted therapy, immunotherapy

## Abstract

**Simple Summary:**

Traditional therapeutic approaches to esophageal adenocarcinoma involve a combination of surgery, chemotherapy, and radiation. Despite innovations in treatment, outcomes remain poor. Targeted molecular therapies and immunotherapies have been used to great effect in various other solid tumors. Several targeted agents show promise in treating esophageal adenocarcinoma. In this review, we aim to highlight recent developments in the arena of targeted therapeutics and suggest topics of future investigations.

**Abstract:**

Esophageal adenocarcinoma is one of the leading causes of cancer-related deaths worldwide. The incidence of esophageal adenocarcinoma has increased at an alarming rate in the Western world and long-term survival remains poor. Current treatment approaches involve a combination of surgery, chemotherapy, and radiotherapy. Unfortunately, standard first-line approaches are met with high rates of recurrence and metastasis. More recent investigations into the distinct molecular composition of these tumors have uncovered key genetic and epigenetic alterations involved in tumorigenesis and progression. These discoveries have driven the development of targeted therapeutic agents in esophageal adenocarcinoma. While many agents have been studied, therapeutics targeting the human epidermal growth factor receptor (HER2) and vascular endothelial growth factor (VEGF) pathways have demonstrated improved survival. More recent advances in immunotherapies have also demonstrated survival advantages with monoclonal antibodies targeting the programmed death ligand 1 (PD-L1). In this review we highlight recent advances of targeted therapies, specifically agents targeting receptor tyrosine kinases, small molecule kinase inhibitors, and immune checkpoint inhibitors. While targeted therapeutics and immunotherapies have significantly improved survival, the benefits are limited to patients whose tumors express biomarkers such as PD-L1 and HER2. Survival remains poor for the remainder of patients with esophageal adenocarcinoma, underscoring the critical need for development of novel treatment strategies.

## 1. Introduction

Esophageal cancer is a highly aggressive malignancy characterized by exceedingly poor survival. In 2018, there were 572,034 newly diagnosed cases of esophageal cancer worldwide, with 508,585 deaths [1]. Esophageal malignancy is primarily comprised of two distinct histological entities, i.e., esophageal squamous cell carcinoma (ESCC) and esophageal adenocarcinoma (EAC). Worldwide, ESCC is more prevalent while EAC dominates in Western countries. In 2020, is has been estimated that there could be 18,440 newly diagnosed cases of esophageal cancer in the United States with an estimated 16,170 deaths. The current five-year survival of esophageal cancer at any stage is 20% which has not changed significantly over the past 15 years. Five-year survival drops to approximately 5% in stage III or IV disease [2,3]. Over the past three decades, there has been a sustained increase in the incidence of EAC in many Western countries. In fact, EAC is the most rapidly increasing form of cancer in certain demographic subpopulations [4]. Males are significantly more frequently affected, and most cases occur in patients over 60 years old. This significant increase in EAC is thought to be due to the population-wide rise of its primary chronic risk factors, i.e., gastrointestinal reflux disease (GERD) and obesity [5]. The reflux of bile acids and gastric contents into the esophagus triggers a metaplastic response in the esophageal epithelium termed Barrett’s esophagus (BE). The key factor defining BE is the replacement of the normal squamous cells lining of the esophagus with a metaplastic columnar epithelium that contains goblet cells [6]. Patients diagnosed with BE are 11 times more likely to develop EAC, representing a yearly incidence from 0.12% to 0.36% [7]. While GERD and BE are well established risk factors in the development of EAC, malignancy can arise without either being present. Up to 40% of patients diagnosed with EAC do not have reflux symptoms and a systematic review found that only 24% to 64% of resected EAC specimens had histological evidence of BE at the time of surgery [8,9]. Once detected, BE requires periodic surveillance for the development of EAC [4]. Given the proportion of EAC patients presenting without evidence of BE or GERD, population-wide surveillance for BE remains to be controversial.

The demarcation between gastric and esophageal cancers is an area of ongoing discussion. The 8th edition of the American Joint Committee on Cancer (AJCC) staging guidelines utilized anatomical location to distinguish between gastric adenocarcinoma (GAC) and EAC. Adenocarcinomas with epicenters less than 2 cm into the gastric cardia are staged as EAC, while tumors greater than 2 cm into the gastric cardia are staged as GAC [10]. The anatomical approach to staging remains controversial as it does not consider the molecular differences in tumor types. To better classify gastric and esophageal adenocarcinomas, the Cancer Genome Atlas (TCGA) recently performed molecular profiling of both tumor types [11,12]. Researchers subdivided GAC into the following four distinct molecular subtypes: Epstein-Barr virus (EBV)-positive, microsatellite instability (MSI), genomically stable (GS), and chromosomal instability (CIN) [11]. Investigators noted significant molecular similarity between EAC and the CIN subtype of GAC, challenging the notion that these diseases are distinct entities [12]. These studies represent a broader effort to categorize EAC into distinct subpopulations that may be clinically useful for developing and targeting molecular therapies. Thus, in our review, we aim to provide the most current information regarding clinical trials involving targeted molecular therapies, as well as to suggest directions for future investigations.

## 2. Current Therapies

### 2.1. Surgery

Surgical resection remains to be a critical component of the overall treatment regimen of EAC. The choice of surgical approach depends largely on tumor characteristics and location. Patients with localized disease may be candidates for endoscopic interventions. Current guidelines from the National Comprehensive Cancer Network (NCCN) recommend endoscopic mucosal resection (EMR) for patients with Barrett’s esophagus, Tis (high-grade dysplasia, carcinoma in situ), T1a, and select T1b lesions without lymphovascular invasion [13].

Patients with resectable lesions who do not meet criteria for endoscopic intervention will generally undergo esophagectomy. For patients with stage I or II disease, surgery has been demonstrated to have similar overall survival and R0 (microscopically margin-negative) resection rates as compared with neoadjuvant chemoradiotherapy followed by surgery [14]. The stomach is the preferred alimentary conduit due to its robust and redundant blood supply. In cases where the stomach cannot be safely utilized, a colonic interposition can be performed with good results [15]. The two primary approaches to esophagectomy are the transhiatal or the transthoracic approach. Multiple meta-analysis comparing surgical techniques have not shown a significant difference in the five-year survival between the two approaches, although the transhiatal approach has fewer overall complications with decreased length of hospital stay [16,17].

The minimally invasive esophagectomy (MIE) has become an increasingly popular approach. Comparative results from multi-institutional randomized clinical trials are still needed, however meta-analysis of published data have been able to demonstrate oncologic equivalency between open and minimally invasive approaches [18]. MIE may offer some advantages including reduced in-hospital mortality, shorter hospital stays, enhanced postoperative recovery, and fewer pulmonary and cardiovascular complications [19]. There are multiple ongoing randomized clinical trials comparing MIE to open esophagectomy (ROMIO, ISRCTN 59036820) [20].

### 2.2. Chemotherapy

Numerous phase III randomized clinical trials have evaluated the utility of perioperative chemotherapy and surgery as compared with surgery alone. The MAGIC trial was a landmark study that randomized patients with resectable adenocarcinoma of the stomach, GE junction, and lower esophagus to perioperative epirubicin, cisplatin, 5-fluorouracil (5-FU) and surgery, or surgery alone. There was an improved five-year survival (36% vs. 23%, *p* < 0.001) in perioperative chemotherapy group as compared with surgery alone [21]. The ACCORD-07 and OE02 studies similarly randomized patients to perioperative chemotherapy (cisplatin and 5-FU) and surgery or surgery alone. Both studies demonstrated a significant survival advantage in the perioperative chemotherapy group (ACCORD-07: HR 0.69, *p* = 0.02) (OE02: HR0.79, *p* = 0.004) [22,23]. A six-year follow up of the OE02 patients demonstrated a preserved survival advantage in the perioperative chemotherapy group (HR 0.84) with an absolute five-year survival of 23% vs. 17.1% [24]. The OE05 trial sought to determine whether increasing the duration and intensity of the neoadjuvant chemotherapy regimen would further improve survival. Patients were randomized to receive two cycles of cisplatin and 5-FU or four cycles of epirubicin, cisplatin, and capecitabine. The intensified neoadjuvant schedule showed no improvement in survival (23.4 vs. 26.1 months). There was no difference in chemotherapy toxicity or surgical complications [25]. The current treatment recommendations for perioperative chemotherapy came from the FLOT-4 study. This phase II/III trial randomized patients with resectable gastroesophageal adenocarcinoma to perioperative epirubicin, cisplatin, and 5-FU/capecitabine, or perioperative 5-FU, leucovorin, oxaliplatin, and docetaxel (FLOT). Results demonstrated significantly improved survival in the FLOT group (HR 0.77, *p* = 0.012) and similar rates of adverse events, most frequently anemia, neutropenia, nausea, and peripheral neuropathy [26].

### 2.3. Chemoradiotherapy

The use of perioperative chemotherapy has become widely utilized in the treatment of EAC, and many U.S. institutions are now adopting neoadjuvant chemoradiotherapy as the treatment regimen of choice. The CROSS trial was a landmark study that first demonstrated the potential benefit of concomitant chemotherapy and radiotherapy in resectable disease. Patients with resectable lesions of the esophagus or GE junction were randomized to receive neoadjuvant chemoradiation with carboplatin, paclitaxel, and radiation followed by surgery or surgery alone. The chemoradiation group was found to have significantly increased R0 resection rates (92% vs. 69%, *p* < 0.001) and improved median overall survival (49.4 vs. 24.0 months, *p* < 0.003) [27]. Post hoc analysis confirmed this survival advantage in both histological subtypes (EAC and ESCC) independently [28]. While the CROSS study demonstrated a clear survival advantage with neoadjuvant chemoradiation in resectable EAC, the addition of radiotherapy had certain disadvantages. Neoadjuvant radiotherapy has been demonstrated to increase intraoperative risks from bleeding, edema, fibrosis, and inflammation, to increase the incidence of postpreparative pulmonary complications including ARDS, and to prolong induction treatment time prior to surgical resection [29,30,31,32]. Despite these drawbacks, multiple meta-analyses have demonstrated consistent significant benefits to locoregional control in patients receiving neoadjuvant chemoradiation as compared with chemotherapy alone [33,34,35,36,37].

Chemoradiation can also be used as a definitive therapy for patients with unresectable disease or for those who are unable to tolerate surgery. The landmark RTOG 8501 trial evaluated the potential benefits of definitive chemoradiotherapy in patients with locally advanced, unresectable esophageal cancers. Participants were randomized to receive either radiation or combined radiation plus cisplatin and 5-FU. The study demonstrated a significant five-year survival advantage in the chemoradiation group (26% vs. 0%, *p* < 0.001) [38].

## 3. Targeted Therapies

**Figure 1:**
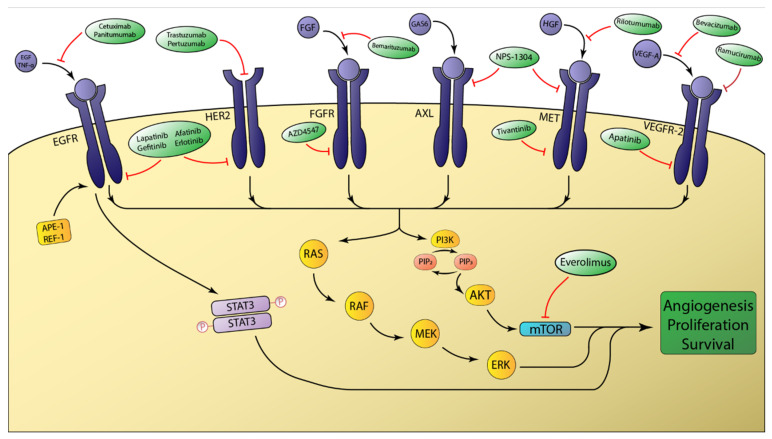
Schematic overview of select receptor tyrosine kinases involved in esophageal adenocarcinoma tumorigenesis. The intracellular signaling of the depicted receptor tyrosine kinases (RTKs) is primarily mediated by the RAS/RAF and PI3K/AKT pathways. Aberrant activation of these pathways ultimately favors tumor growth and survival. mTOR is a major downstream effector that is modulated by AKT signaling. Molecular therapies targeting RTKs either disrupt ligand binding or function to prevent intracellular activation of the tyrosine kinase domain.

Targeted therapies differ from traditional chemotherapy regimens in that they are designed to bind and inhibit specific molecules overexpressed in an individual patient’s tumor. The rationale of targeted molecular therapies is rooted in the concept of “oncogene addiction”. This term was coined as a means of describing the degree by which tumor cells are dependent on individual oncogenes to sustain their malignant phenotype [39]. Despite the various alterations present in cancer cells, inactivation of even a single oncogene can inhibit cellular growth and proliferation. The theory is that activated oncogenes result in a state of hyperproliferative signaling that, when suddenly inactivated, shift the cellular balance towards proliferative arrest and apoptosis [40]. Perhaps the most famous example of oncogene addiction being exploited for targeted therapeutics is in chronic myeloid leukemia (CML). This disease is driven by a chromosomal translocation resulting in the *BCR-ABL* mutant oncogene. Direct inhibition of this oncogene via the small molecule kinase inhibitor imatinib (Gleevec) resulted in a major clinical response in 87% of treated CML patients as compared with just 35% clinical response in a comparison group receiving interferon alfa plus cytarabine (*p* < 0.001) [41]. The concept of oncogene addiction is not universal and targeting certain oncogenes may not result in a complete response. It is important to understand the circumstances by which oncogene inactivation induces apoptosis, differentiation, and senescence in the context of heterogenous and complex activation of multiple signaling networks within a tumor. This knowledge will help to guide development of novel and effective strategies in the treatment of cancer [42]. In the following sections, we will briefly review the current state of targeted molecular therapies in the treatment of EAC (Figure 1).

### 3.1. The ErbB Receptor Family

The erbB family comprises a group of tyrosine kinase receptors that integrate external stimuli with specific internal signals that allow cells to appropriately respond to the environment. Overexpression and aberrant function of receptors belonging to this family have been frequently implicated in many human cancers. This family contains the following four distinct receptors: erbB1 (EGFR/HER1), erbB2 (HER2/Neu), erbB3 (HER3), erbB4 (HER4). These receptors share the same molecular structure with an extracellular binding domain, a short transmembrane domain, and an intracellular domain. Except for HER3, the intracellular domain has tyrosine kinase activity [43]. The formation of erbB homo and heterodimers upon ligand binding activates the intrinsic receptor tyrosine kinase (RTK) activity via intramolecular phosphorylation and generates downstream signaling effects via activation of the RAS/RAF and PI3K/AKT pathways (Figure 1) [44].

### 3.2. Epidermal Growth Factor Receptor

The epidermal growth factor receptor (EGFR) plays an integral role in normal cell growth and differentiation. Its primary ligands are EGF and TGF-α [44]. Aberrant activation of EGFR via amplification, overexpression, or mutation has been demonstrated to induce tumor cell proliferation, migration, and metastasis. EGFR has been found to be preferentially amplified in patients with poorly differentiated EAC [45]. The overall prevalence of EGFR amplification in EAC has been reported to be between 4 and 6% [46]. EGFR has been shown to be a key mediator in the neoplastic progression to EAC. Apurinic/apyrimidinic endonuclease 1 (APE1) is constitutively overexpressed in EAC and can be induced in non-neoplastic esophageal tissue after exposure to acidic bile salts (ABS). APE1 induction activates EGFR which subsequently phosphorylates and activates STAT3. The ultimate consequence of APE1-EGFR-STAT3 axis activation in EAC is constitutive STAT3 signaling which results in a survival advantage and promoted cell proliferation (Figure 1) [47]. Caudal homeobox 2 (CDX2) induction has also been implicated in the metaplastic transition to Barrett’s esophagus [48]. The mechanism by which ABS induces CDX2 expression is through ligand-dependent transactivation of EGFR [49]. EGFR plays an integral role in EAC tumorigenesis and, as such, has been the subject of investigation for targeted molecular therapies.

Cetuximab and panitumumab are monoclonal antibodies directed against EGFR and both are currently FDA approved for the treatment of *KRAS*-wild type metastatic colorectal cancer [50]. A recent phase III clinical trial evaluated the utility of cetuximab in patients with locally advanced, unresectable, or metastatic gastroesophageal adenocarcinoma. Patients were randomized to receive standard chemotherapy with or without cetuximab. Surprisingly, the addition of cetuximab resulted in worse progression-free survival (4.4 vs. 5.6 months) [51]. A separate phase III trial evaluating panitumumab also demonstrated worse overall survival in a similar patient population [52].

Small molecule tyrosine kinase inhibitors are a separate class of targeted therapeutics that competitively bind the ATP binding site of the intracellular tyrosine kinase domain of EGFR and other tyrosine kinase receptors. This prevents intracellular signal transduction even in the presence of bound ligand. Gefitinib is a small molecule inhibitor of HER2 and EGFR. Preclinical studies have demonstrated good antitumor activity of gefitinib in EAC cell lines, especially those with EGFR polysomy [53]. The COG trial was a randomized phase III trial studying gefitinib in patients with chemoresistant EAC and ESCC. There was no survival difference in patients receiving gefitinib or placebo (3.73 vs. 3.67 months) [54]. Subgroup analysis, however, demonstrated that, in the 20.2% of enrolled patients who had EGFR copy number gain (CNG) demonstrated by fluorescent in situ hybridization (FISH), there was significantly improved overall survival with gefitinib (HR 0.59, *p* = 0.05). Those patients with EGFR amplification (7.2%) benefited the most from gefitinib therapy (HR 0.21, *p* = 0.006) [55]. More recent investigations have mirrored the COG subgroup analysis and evaluated whether EGFR CNG could be a biomarker predictive of response to anti-EGFR therapy [56]. Post hoc analysis of multiple larger clinical trials has uncovered an association between the EGFR amplified subpopulation and overall survival [55,57]. In a large single-intuition cohort of patients with metastatic gastroesophageal adenocarcinoma with EGFR amplification, anti-EGFR therapy was found to provide an excellent clinical response in a majority of patients [58]. Larger studies validating the EGFR CNG biomarker may uncover some benefit in this class of targeted therapeutics.

### 3.3. Human Epidermal Growth Factor Receptor 2

The human epidermal growth factor receptor 2 (HER2) is the second member of the erbB family of receptors and shows significant homology to EGFR. Interestingly, HER2 is the only member of this receptor family that has no identified natural ligand and often heterodimerizes with other erbB receptors [44]. Heterodimers that form with HER2 generate intracellular signals that are significantly more robust than other erbB receptors [59]. HER2 signaling is involved in various cellular functions including cell growth, survival, and differentiation. The primary HER2-mediated signal transduction pathways involve mitogen-activated protein kinase (MAPK) and phosphatidylinositol 3-kinase (PI3K) [60]. HER2 amplification has been demonstrated to be a poor prognostic indicator and oncogenic amplifications of HER2 have been reported in 20–30% of EAC cases as compared with just 3% in ESCC [12,61].

Trastuzumab is a humanized monoclonal antibody targeting the extracellular domain of HER2 that has become the standard of care for patients with HER2-positive breast cancer [62]. The trastuzumab for gastric cancer (TOGA) trial evaluated patients with advanced gastroesophageal adenocarcinoma with confirmed overexpression of HER2 (via FISH or IHC). Participants were randomized to receive standard chemotherapy with or without trastuzumab. The results demonstrated significantly improved overall survival (13.8 vs. 11.1 months, *p* = 0.0046) in the trastuzumab group [63]. Unfortunately, this survival advantage was not observed in a recently reported phase III trial evaluating the addition of trastuzumab to neoadjuvant chemoradiation in the setting of resectable, HER2-positive EAC [64].

Pertuzumab is another humanized monoclonal antibody targeting HER2 that was specifically developed to prevent HER2 dimerization. Pertuzumab binds to a different epitope on the extracellular domain of HER2 and functions to sterically block HER2 dimerization [65]. Preclinical studies have demonstrated synergistic antitumor activity when combined with trastuzumab [66,67]. In HER2-positive metastatic breast cancer, phase III clinical trials have demonstrated significantly longer progression-free survival in patients receiving the combination of pertuzumab and trastuzumab [68]. A similar phase III trial was conducted to evaluate the use of dual HER2 therapy in patients with HER2-positive, metastatic gastroesophageal adenocarcinoma. Patients were treated with trastuzumab and chemotherapy with either pertuzumab or placebo. The results demonstrated a modest but not statically significant improvement in overall survival with the addition of pertuzumab (17.5 vs. 14.2 months, *p* = 0.057) [69]. A recently published phase II study evaluated the feasibility of adding pertuzumab and trastuzumab to standard neoadjuvant chemoradiotherapy in patients with resectable, HER2-positive EAC. Investigators demonstrated the feasibility of dual HER2 blockade through exploratory propensity score-matched comparisons to a cohort receiving standard neoadjuvant chemoradiotherapy and demonstrated significantly longer overall survival (HR 0.58, *p* < 0.05) with dual anti-HER2 therapy [70]. Larger phase III trials will be needed to determine if targeted dual HER2 therapy has a role in the neoadjuvant setting for the treatment patients with resectable disease.

Unfortunately, trastuzumab’s clinical effect in gastroesophageal adenocarcinoma has been modest as primary resistance is common and acquired resistance develops quickly. A recent phase II study characterized tumors resistant to targeted therapy. Investigators uncovered an association between co-amplification of EGFR and HER2 and better clinical response to targeted HER2 therapy [71]. Further investigation is warranted to determine if differential expression of HER2 and EGFR can predict clinical response to targeted therapies in HER2-amplified EAC.

### 3.4. Vascular Endothelial Growth Factor

The interactions between vascular endothelial growth factors (VEGF) and VEGF receptors (VEGFR) are the principle means for regulation of angiogenesis. These interactions play a key role in tumor growth and metastasis [72]. VEGF-A is the primary ligand involved in tumor angiogenesis and binds with high affinity to VEGFR-1 and VEGFR-2, although most proangiogenic effects are mediated by VEGFR-2 [73]. VEGF is commonly elevated in solid tumors due to intra-tumoral hypoxia. Hypoxia induces VEGF via hypoxia inducible factors (HIF) 1α and 2α [74]. The angiogenic properties of EAC are often acquired early in the tumorigenesis of EAC. While VEGF expression has been associated with tumor progression and metastasis in ESCC, there has been little evidence of similar prognostic indication in EAC [75]. A study of the Barrett’s adenocarcinoma cell line OE33 demonstrated increased VEGF expression following exposure to ABS [76]. This upregulation of VEGF suggests that acid reflux has the potential to contribute to tumorigenesis via angiogenesis.

Bevacizumab is a monoclonal antibody directed against VEGF-A. It has been FDA approved for the treatment of various solid tumors including metastatic colorectal cancer, glioblastoma, non-small cell lung cancer, and cervical cancer [77]. In phase III trials, the addition of bevacizumab to capecitabine and cisplatin in patients with advanced gastroesophageal adenocarcinoma demonstrated no significant improvement in overall survival (12.1 vs. 10.1 months, *p* = 0.1002). While the primary endpoint was not met, the study did reveal significantly improved progression-free survival (6.7 vs. 5.3 months, *p* = 0.0037) and overall clinical response rate (46.0% vs. 37.4%, *p* = 0.0315) for patients receiving bevacizumab [78]. A phase II/III trial evaluating the addition of bevacizumab to a perioperative chemotherapy regimen demonstrated no significant increase in three-year overall survival (50.3% vs. 48.1%, *p* = 0.36), higher incidence of wound healing complications (12% vs. 7%), and higher anastomotic leak rates (24% vs. 10%) with the addition of bevacizumab [79].

Ramucirumab is a newer IgG1 monoclonal antibody targeting VEGFR-2 that showed robust activity in the preclinical setting [80]. A phase III trial evaluating patients with advanced, chemoresistant gastroesophageal adenocarcinoma randomized patients to receive ramucirumab or placebo. There was a significant overall survival advantage (5.2 vs. 3.8 months, *p* = 0.047) in the ramucirumab group [81]. A separate study evaluating the same patient population randomized participants to receive second-line therapy with paclitaxel and ramucirumab or paclitaxel alone. Overall survival was again found to be significantly longer in the ramucirumab group (9.6 vs. 7.4 months, *p* = 0.017) [82]. These trials resulted in the FDA approving ramucirumab in combination with paclitaxel as a second-line therapy for the treatment of advanced gastroesophageal adenocarcinoma [83]. More recent studies evaluating ramucirumab as a first-line agent have been less successful. Patients with HER2-negative gastroesophageal adenocarcinoma were randomized to receive cisplatin plus capecitabine with either ramucirumab or placebo. The results demonstrated no significant improvement in progression-free survival or overall survival [84].

Apatinib is a small molecule inhibitor that is highly selective for VEGF-2. A recent phase III trial evaluated its utility in treating patients with advanced, chemoresistant gastroesophageal adenocarcinoma. Results demonstrated significantly prolonged median overall survival (6.5 vs 4.7 months, *p* = 0.0149) and progression-free survival (2.6 vs 1.8 months, *p* < 0.001) in patients receiving apatinib compared to placebo [85].

VEGF-targeted therapy appears to have a role in the overall treatment paradigm of EAC. There are multiple ongoing clinical trials attempting to identify the setting in which VEGF therapy would be most beneficial. The postoperative complications associated with VEGF-targeted therapy, have limited its utility to patients with advanced, unresectable disease, although smaller studies have suggested these adverse postoperative effects may be less severe with apatinib [86]. Further investigations are warranted to discover if apatinib has a place in the treatment of resectable disease.

### 3.5. Mesenchymal-Epithelial Transition Factor

The mesenchymal-epithelial transition (*MET*) proto-oncogene is often implicated in a multitude of cancers and plays a role in tumorigenesis and metastasis. *MET* encodes the receptor tyrosine kinase MET (or cellular MET, c-MET). Its ligand, hepatocyte growth factor (HGF), is primarily secreted by cells of mesenchymal origin [87]. MET activation triggers diverse signaling pathways that are primarily mediated by the RAS/RAF and PI3K/AKT pathways [88]. Aberrant MET activation plays a critical role in tumor cell proliferation, invasion, and angiogenesis [89]. Genomic amplification of MET has been demonstrated to be a poor prognostic indicator in EAC [90,91].

Our understanding of the role MET plays in tumor biology has made it an attractive target in the development of novel therapeutics. Unfortunately, most phase III clinical trials have been unable to demonstrate a role for targeted MET inhibition in the treatment of EAC. Rilotumumab is a monoclonal antibody targeting HGF. A phase III trial evaluating rilotumumab in patients with MET-positive gastroesophageal adenocarcinoma was ended early after results demonstrated worse median overall survival (8.8 vs. 10.7 months) with the addition of rilotumumab to standard chemotherapy [92]. Onartuzumab is another monoclonal antibody targeting the extracellular domain of MET to prevent HGF binding. A phase III trial evaluating the addition of onartuzumab to standard first line mFOLFOX6 in patients with HER2-negative, MET-positive gastroesophageal adenocarcinoma demonstrated no improvement in overall survival (11.0 vs. 11.3 months) or progression-free survival (6.7 vs. 6.8 months) [93]. While trials involving anti-MET antibodies have been unsuccessful, MET small molecule inhibitors have shown promising results. Tivantinib is one such small molecule inhibitor that preferentially interacts with tumor cells overexpressing MET [94]. A phase II trial evaluating the addition of tivantinib to standard FOLFOX for metastatic gastroesophageal adenocarcinoma showed improved progression-free survival as compared with historical controls [95]. While these findings are encouraging, further phase III are warranted to validate the results. Targeted MET therapy may also be a means of overcoming resistance to other targeted therapeutics. For example, a study of HER2 resistant tumors uncovered an association between resistance and MET amplification. Patient-derived xenograft models, established from these co-amplified tumors, demonstrated complete tumor regression with dual HER/MET inhibition [71]. Further investigation is needed to determine if the observed preclinical effect of combined HER2/MET inhibition is a viable strategy to overcome HER2 resistance.

### 3.6. Fibroblast Growth Factor Receptor

The fibroblast growth factor receptor (FGFR) family is another RTK known to be involved in tumor growth, survival, resistance to chemotherapeutic regimens, and angiogenesis [96]. To date, four distinct FGFR receptors have been identified (FGFR1–4). Similar to many other RTKs, interaction of FGFR with its ligand triggers an intracellular signaling cascade primarily through PI3K/AKT and RAS/RAF pathways [97]. Genomic amplification of FGFR1 and FGFR2 has been reported in 3–4% of EAC samples and appeared to confer a poor prognostic in gastric adenocarcinoma [12,98].

Bemarituzumab is a humanized monoclonal antibody targeting FGFR2b. Mechanistically, bemarituzumab inhibits ligand binding and induces internalization and degradation of FGFR2b [99]. Phase I and II trials, evaluating the utility of bemarituzumab in treating FGFR2b-overexpressed (via IHC or circulating tumor DNA) advanced gastric cancers, demonstrated adequate safety profiles and significant antitumor activity [100]. Planned phase III trials are underway to evaluate if the addition of bemarituzumab to standard FOLFOX6 chemotherapy is beneficial in this subset of FGFR2b amplified tumors [99].

AZD4547 is a highly selective small molecule tyrosine kinase inhibitor of FGFR1–3. Preclinical studies have shown good results in FGFR2 amplified cell lines and mouse models [101]. A recent phase II trial selected patients with advanced gastric adenocarcinoma and FGFR2 polysomy or gene amplification (via FISH) to receive AZD4547 or paclitaxel. The results demonstrated no significant improvement in progression-free survival. Exploratory biomarker analysis of FGFR2 amplification/polysomy demonstrated poor concordance with measured FGFR2 mRNA expression and significant intra-tumoral heterogeneity [102]. The inability to select for clonally amplified tumors may explain why the preclinical efficacy of AZD4547 was unable to be translated in clinical trials. Perhaps utilization of circulating tumor DNA, as a means of selecting the appropriate study population, will lead to better results in future investigations.

FGFR2 plays a critical role in angiogenesis by promoting proliferation and migration of vascular endothelial cells, especially in combination with VEGF [103]. A phase II study evaluating biomarkers for resistance to anti-VEGF therapy in metastatic colorectal cancer found significantly increased FGFR expression [104]. Multiple studies have demonstrated enhanced anti-angiogenic effects with dual FGFR/VEGF therapy in solid tumors [105,106]. The combination of dual inhibition of VEGF and FGFR has yet to be evaluated in EAC and is a promising area of future studies.

### 3.7. Mammalian Target of Rapamycin

The mammalian target of rapamycin (mTOR) is a master regulator of cellular growth, proliferation, and survival [107]. Many RTKs achieve downstream effects through signaling primarily mediated by the RAS/RAF and PI3K/AKT pathways. mTOR is downstream from these signaling pathways and is one of the major effectors regulated by PI3K/AKT signaling [108]. Aberrant regulation of mTOR has been proposed as a potential mechanism for the ineffectiveness of previously mentioned RTK-targeted therapies [109].

Everolimus is an oral mTOR inhibitor that has been approved for the treatment of advanced renal cell carcinoma and advanced breast cancer [110,111]. Preclinical studies have demonstrated significant tumor activity of everolimus in gastric cancer cell lines [112]. Phase III trials of patients with advanced gastroesophageal adenocarcinoma demonstrated modest, but not significant improvement in overall survival with the addition of everolimus to standard chemotherapy (5.4 vs. 4.3 months, *p* = 0.124) [113]. A more recent phase II trial identified pS6 as a potential biomarker in patients with advanced gastroesophageal adenocarcinoma who responded to everolimus [114]. Further investigations are needed to validate pS6 as a positive predictor marker and to uncover other potential biomarkers to stratify patients who would best respond to targeted mTOR therapy.

### 3.8. Heat Shock Protein 90

Heat shock proteins (HSP) are a family of molecular chaperones that are often a cellular response to stressful conditions. They function to prevent aggregation of misfolded proteins by ubiquitin-targeted degradation and to assist proper protein folding [115]. HSP90 is a member of this molecular chaperone family and is overexpressed in EAC [116]. In cancer cells, HSP90 can act to stabilize mutated or overexpressed oncogenic client proteins [117]. Targeted therapy allows HSP90 to release bound client proteins which can subsequently be degraded [118].

HER2 is an HSP90 client protein that has been the subject of recent investigations. A study of human EAC samples demonstrated a strong positive correlation between HSP90 expression and HER2 status, suggesting coregulation in a subset of EAC patients [119]. In fact, some data suggest that dysregulated HSP90 expression may be responsible in potentiating the oncogenic effects of HER2, representing a potential mechanism of resistance to HER2-targeted therapies [120]. Studies investigating the effects of combined HER-2 and HSP90 inhibition in trastuzumab or lapatinib-resistant gastric cancer models have shown significant synergistic antitumor activity [121,122]. While clinical trials studying combined therapy in EAC are lacking, a published phase II trial found that a majority (69%) of patients with metastatic breast cancer, treated with the combination of the HSP90 inhibitor AUY992 and trastuzumab, achieved clinical response or stable disease [123]. In EAC models, AUY992 has demonstrated robust antitumor activity when combined with 5-FU and cisplatin [124]. An ongoing phase II clinical trial is evaluating the effects of AUY922 with docetaxel and irinotecan in patients with advanced gastroesophageal adenocarcinoma (NCT 01084330). Ganetespib is a recently developed small molecule inhibitor of HSP90 that is currently being investigated in a phase I clinical trial (NCT 02389751) in combination with carboplatin, paclitaxel, and radiation for patients with stage II–III EAC. Targeted HSP90 therapy may provide a means to overcome resistance to previously mentioned RTK-targeted therapeutics.

### 3.9. Aurora Kinase A

**Figure 2:**
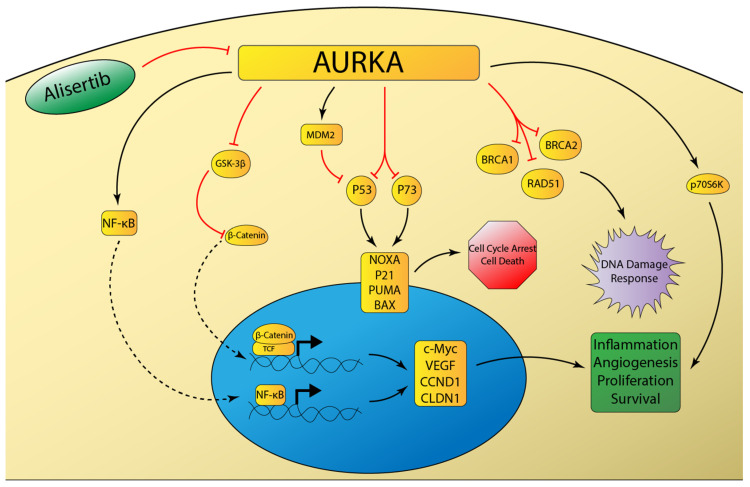
Schematic representation of aurora kinase A (AURKA) signaling networks involved in cancer. AURKA overexpression inhibits the DNA damage response and attenuates DNA-damage induced cell cycle arrest and apoptosis through inhibition of p53 family member proteins [129]. AURKA induces inflammatory signaling mediated by NF-κB [133], as well as pro-survival signaling mediated by beta-catenin [132] and p70S6K [135].

Aurora kinase A (AURKA) is one of three mammalian serine-threonine protein kinases in the aurora kinase family. AURKA plays a critical role in mitotic spindle formation and centrosome development. The *AURKA* gene is located on chromosome 20q13, a locus that is frequently amplified in gastroesophageal adenocarcinoma [125]. Preclinical studies have demonstrated AURKA overexpression to impair DNA damage repair through inhibition of RAD51, BRCA1, and BRCA2 [126,127,128] (Figure 2). AURKA also significantly inhibits the tumor-suppressive functions of p53 and p73 [129]. The inhibition of p53 occurs by the following two distinct mechanisms: MDM2-mediated degradation and direct phosphorylation at Ser315 and Ser215 [130,131]. Furthermore, AURKA overexpression leads to phosphorylation of GSK-3β, which in turn decreases phosphorylation of beta-catenin and ultimately results in increased oncogenic signaling of the beta-catenin/TCF transcription complex [132]. Moreover, AURKA can induce inflammation in gastric cancer cells through induction of NF-κB [133]. Because of its intimate role in tumorigenesis, AURKA-targeted therapy has been the focus of recent drug development. AURKA therapy in EAC is particularly interesting, due to the abundance of p53 mutations or deficiencies in human tumors [134].

The identification of AURKA as a druggable target has spurred the development of many investigational agents targeting AURKA. Alisertib (MLN8237) is a selective inhibitor of AURKA and has been shown in preclinical studies to induce mitotic spindle abnormalities, mitotic accumulation, and apoptosis [136]. Studies of EAC models have demonstrated robust antitumor activity¸ especially when combined with cisplatin and docetaxel [137,138]. A recent phase I/II trial evaluated the antitumor activity of alisertib in a variety of advanced solid tumors including breast cancer, lung cancer, and gastroesophageal adenocarcinoma. The results demonstrated a manageable safety profile and promising antitumor activity, although this effect was less pronounced in the gastroesophageal cohort [139]. The frequency of AURKA overexpression in EAC makes it an attractive target for further drug development and clinical trials. While preclinical models have shown promising results, further trials are needed to determine if AURKA inhibition provides real clinical benefit.

### 3.10. AXL

The *AXL* gene encodes a receptor tyrosine kinase in the TAM family [140]. Upon interaction with its ligand, GAS6 (growth arrest-specific gene 6), AXL results in activation of the PI3K/AKT and RAS/RAF pathways. Aberrant activation of these pathways drives angiogenesis, proliferation, survival, invasiveness, and the epithelial to mesenchymal (EMT) transition [141]. AXL overexpression is a poor prognostic indicator in EAC [142]. Preclinical studies have demonstrated that aberrant AXL expression was intimately involved in the resistance of EAC to standard chemotherapeutic regimens. AXL was found to mediate resistance to cisplatin through disruption of c-ABL nuclear accumulation in response to DNA damage [143]. Additionally, AXL can promote resistance to epirubicin via transcriptional upregulation of c-MYC. In fact, AXL knockdown or pharmacologic AXL inhibition sensitizes resistant cells to epirubicin [144]. Preclinical investigations have also demonstrated that AXL activation mediated resistance to EGFR-targeted therapeutics in lung cancer [145]. Subsequent studies have demonstrated that the AXL inhibitor NPS-1034 was effective in rendering these cells sensitive to EGFR-therapy [146]. While further investigation is warranted, AXL blockade may be exploited to sensitize EAC cells to standard chemotherapy regimens or to targeted EGFR therapeutics.

## 4. Immune Checkpoint Inhibitors

**Figure 3:**
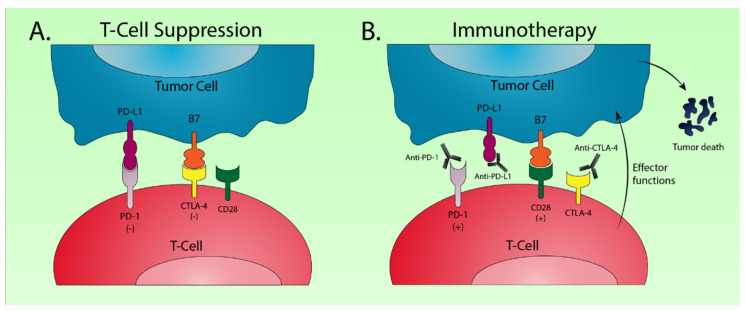
Schematic drawing of the major molecular mechanisms involved in checkpoint blockade for cancer immunotherapy. (**A**) Interaction of the programmed death 1 (PD-1) receptor on T-cells with the programmed death ligand 1 (PD-L1) on tumor cells results in T-cell suppression [148]. A similar suppressive interaction occurs between B7 on tumor cells and cytotoxic T lymphocyte-associated antigen 4 (CTLA-4) on T-cells [149]; (**B**) Monoclonal antibodies targeting PD-1 and PD-L1 disrupt T-cell checkpoint suppression and facilitate antitumor T-cell effector functions. Anti-CTLA-4 therapy allows binding of B7 with CD28 on T-cells which stimulates further T-cell activation.

The T-cell immune response is a tightly regulated process that functions by costimulatory and coinhibitory mechanisms to identify foreign antigens and protect the host from autoimmunity. Cancer cells will often express various tumor-specific antigens that allow T-cells to recognize the tumor as foreign and induce cell death via apoptosis. Cancer cells, however, can escape immune recognition by expressing coinhibitory signals known as immune checkpoints [147] (Figure 3). Programmed death 1 (PD-1) is a receptor expressed on T-cells that functions primarily to maintain peripheral tolerance by inhibiting T-cell activation. Its ligand, PD-L1, is often overexpressed in cancer cells which allows the tumor to escape immune surveillance [148]. Cytotoxic T lymphocyte-associated antigen 4 (CTLA-4) is another coinhibitory immune checkpoint that suppresses T-cell activation. A critical step in T-cell activation involves the binding of the B7 ligand of tumor cells to the CD28 receptor of T-cells. CLTA-4 functions to inhibit T-cell activity by competing with CD28 for B7, for which CTLA-4 has a significantly higher affinity [149].

Cancer immunotherapy utilizes monoclonal antibodies directed against immune checkpoint proteins such as PD-1, PD-L1, and CTLA-4 to enable T-cell recognition of tumor cells, and delivers the antitumor immune response. Molecular characterization of gastroesophageal adenocarcinoma has uncovered significant immune dysregulation in EBV and MSI subtypes [11]. In fact, high MSI was predictive of solid tumor response to PD-1 inhibition [150]. Pembrolizumab is a monoclonal antibody that binds to PD-1. It was granted FDA approval as a third line therapy in advanced gastroesophageal adenocarcinoma expressing PD-L1 with high MSI or deficient mismatch repair (MMR) [151]. More recently, pembrolizumab has been investigated as a potential first-line option. KEYNOTE-062 (NCT 02494583) is an ongoing phase III study which randomized patients with advanced gastroesophageal adenocarcinoma to receive pembrolizumab, pembrolizumab plus chemotherapy, or chemotherapy alone. Preliminary results demonstrated non-inferiority of single agent pembrolizumab as compared with standard chemotherapy. Pembrolizumab monotherapy also improved median overall survival (17.4 vs. 10.8 months) in patients with high PD-L1 expression [152]. While researchers could not claim statistical significance due to study design, it appeared that pembrolizumab was superior to standard chemotherapy in this subset of patients. Nivolumab is another monoclonal antibody targeting PD-1 that has been investigated in phase III trials, both as a salvage therapy and a first line agent. Patients with advanced gastroesophageal adenocarcinoma who had failed previous chemotherapy regimens demonstrated significantly improved overall survival (5.3 vs. 4.1 months, *p* < 0.0001) with nivolumab as compared with the placebo [153]. Recently reported results from the CheckMate 649 trial show significantly improved overall survival (14.4 vs. 11.1 months, *p* < 0.0001) and progression-free survival (7.7 vs. 6.1 months, *p* < 0.0001) when adding nivolumab to standard first line chemotherapy in patients with advanced gastroesophageal adenocarcinoma and high PD-L1 expression. Interestingly, trial participants were not selected for PD-L1 expression upon enrolment and a significant overall survival advantage (13.8 vs. 11.6 months, *p* = 0.0002) was demonstrated in the entire study population regardless of PD-L1 expression [154].

Dual immune checkpoint blockade is an emerging and exciting area of investigation. Ipilimumab is a monoclonal antibody, which targets CTLA-4, that has been combined with nivolumab to achieve significant survival benefits in metastatic melanoma, small-cell lung cancer, and MMR deficient/high MSI colorectal cancer [155,156,157]. A Phase II trial demonstrated significant antitumor activity and durable response with combined ipilimumab and nivolumab therapy in patients with advanced gastroesophageal adenocarcinoma [158]. Larger phase III trials are currently underway evaluating this drug combination in both advanced (NCT 03143153) and resectable (NCT 03604991) EAC.

## 5. Conclusions

While significant strides have been made in the treatment of EAC, survival remains poor and therapeutic advancements have not kept pace with other GI malignancies. The ability to readily perform molecular profiling has ushered in a new wave of investigations into targeted molecular therapeutics and immunotherapies. Current first-line treatments of EAC are often met with therapeutic resistance. Molecular profiling of these resistant tumors has helped investigators to identify differentially expressed biomarkers that can be exploited to overcome resistance. Many of these mechanisms have only been described in a preclinical setting and are yet to be applied in clinical trials. The next five years will likely see a dramatic shift in the overall treatment paradigm of EAC, especially when it comes to tumors resistant to first-line agents.

First-line therapies will also likely change as investigations into immunotherapies continue. Recent studies are even challenging the notion that positive biomarkers such as PD-L1 are necessary to observe a clinical benefit with anti-PD-L1 immunotherapies. Further improvements in immunotherapies and the identification of druggable targets will likely result from a more in-depth understanding of molecular signatures and tumorigenesis. Clinicians will likely need to revisit the idea of tailoring first-line treatment regiments based on tumor stage, instead of focusing on the molecular profile.
